# Minimum Number of Settlers for Survival on Another Planet

**DOI:** 10.1038/s41598-020-66740-0

**Published:** 2020-06-16

**Authors:** Jean-Marc Salotti

**Affiliations:** 1 Univ. Bordeaux, CNRS, Bordeaux INP, IMS, UMR 5218, F-33400 Talence, France; 2 INRIA, IMS, UMR 5218, F-33400 Talence, France; 3 Association Planète Mars, F-33400 Talence, France

**Keywords:** Astronomy and planetary science, Engineering

## Abstract

What is the feasibility of survival on another planet and being self-sustaining? This question is of particular importance for the future of the space conquest and perhaps also for the future of humanity in general [1,2]. The use of *in situ* resources and different social organizations have been proposed [3–6,12–19] but there is still a poor understanding of the problem’s variables. I show here that a mathematical model can be used to determine the minimum number of settlers and the way of life for survival on another planet, using Mars as the example [6,15]. It is based on the comparison between the time requirements to implement all kinds of human activities for long term survival and the available time of the settlers. An important parameter of the model is called the sharing factor, which allows some reduction of time requirements per individual if, for example, the activity concerns the construction of an object that can be shared by several individuals. For survival on Mars, some assumptions are made for the organization of the settlers and engineering issues [13–15]. The minimum number of settlers has been calculated and the result is 110 individuals. Other assumptions can be made. The proposed method allows assessments and comparisons, opening the debate for the best strategy for survival. If this relatively low number is confirmed, survival on another planet might be easier than expected, provided that the organization of the settlers is appropriate.

## Introduction

Humanity could be threatened with extinction due to some cataclysmic event^1^. As pointed out by Sagan, in this case, the only possible way to avoid the end of the world as we know it might be to settle another planet within a short period of time^2^. But what is the feasibility of survival on another planet for a small group of humans? The problem has already been addressed in terms of sufficient genetic diversity and social adaptation by several authors^3–7^. Marin and Beluffi showed that earlier studies were too constrained^6^. They performed simulations with different multi-generational population sizes and determined on a statistical basis that the group of humans could reasonably survive if the initial size were to be greater than 98. However, the problem has not been addressed in terms of engineering capacities and human resources for long-term survival.

Considering time and payload constraints, the number of people that could be sent to another planet would be rather limited. Space X is currently working on a giant reusable interplanetary vehicle with the objective of sending 100 persons at a time to the red planet. However, this is an optimistic estimate of the capability, the feasibility of the reusability remains uncertain and the qualification of the vehicle for landing on Mars and relaunch from Mars could be very difficult and take several decades. In most well studied mission to Mars scenarios, which last at least 6 months for the outbound trip and for crews of 3 to 6 astronauts, it is suggested to use several giant rockets to carry only a few dozens of tons of consumables to the red planet^8–11^. While on the planet, many authors proposed a settlement process based on the utilization of resources that can be found in the atmosphere or in the soil of the planet^12–15^. For instance, Zubrin proposed to extract carbon dioxide from the Martian atmosphere and water ice from the soil to produce oxygen and organic compounds, hematite to produce iron, silicates to produce glass, etc.^13^. Even if the feasibility of the approach is generally acknowledged, the complexity of the implementation is poorly understood and the number of items that would remain to be sent each year would still represent a tremendous challenge. From a practical point of view, it is not clear how many years it would take, as a minimum, to achieve a reasonable level of self-sufficiency, how many rockets would be required to send resources and goods and what would be the way of life and the organization of the society during the development period. If a slow settlement process is already a great challenge, what if time and payloads were constrained and the objective was the long term survival of the group without help from Earthlings?

Similar issues arise for interstellar travel. In the pioneering works from O’Neill, Bond and Martin, or Matlof, the long term survival of a small group of humans has been addressed^16,17,18^. Engineering and human factor issues were highlighted, especially the reliability of systems, knowledge management and psychology but the feasibility remained uncertain and not well understood. An important question, which is addressed in this paper, is to determine the minimum number of individuals in terms of human resources and the most appropriate organization to survive on an extra-terrestrial world, which might eventually be artificial. In a more recent paper, Hein *et al*. revisited the issue of world ships for interstellar travel^19^. Interestingly, the last words of their conclusion were: “All issues related to determining an adequate crew size will have a major impact on the feasibility of a world ship”. It should be noticed that this question is also of interest if the objective is to settle on a given planet according to a slow and well-established plan. Indeed, there exist many possible dramatic reasons that may cause the failure of such a plan. Suppose for instance that a settlement has already started and that there are regular space trips towards the planet. In case of war on Earth, important space sector infrastructures may be destroyed, causing a long term interruption in space travel. It could also happen that a conflict occurs between the terrestrial governments and the settlers and, later on, a group declares independence and tries to survive on its own. Another reason could be the will of a new government to stop the settlement process because of the never-ending increasing cost.

For all these reasons, the problem of determining the minimum number of individuals for survival is of interest. It is possible to make assumptions and to discuss different scenarios but what is generally lacking is a methodology to perform quantitative assessments, especially for engineering issues. The objective of the paper is to show that mathematical modelling of the problem is possible. In next paragraphs, a mathematical expression is proposed and the parameters are discussed. A case study is also presented concerning survival on Mars. Some assumptions are made and the minimum number of individuals to survive on Mars is determined.

## Mathematical Model

### Main principles

The problem can be mathematically defined in different ways. Basically, regarding human resources, the minimum number of individuals for survival depends on their capacity to produce essential objects and consumables using local resources. The initial state of the settlement is very important because large quantities of resources and modern tools may help a lot in developing industries and achieving a viable state. However, there are two reasons suggesting that the impact of the initial state is not so important. On the one hand, a limiting factor is the cost and complexity of interplanetary trips. On the other hand, the lifetime of modern tools such as vehicles, computers, robots, etc. is in general in the order of ten years, which is very short and implies finding quickly solutions to replace them. For the sake of simplicity, it is assumed here that the initial amount of resources and tools sent from Earth will be rather limited and as a consequence will not have much impact on survival. Providing that the initial state is viable, it is assumed that survival depends only on two important variables:

- Available local resources. A local resource can be a gas, mineral or liquid present on the surface of the planet. It is available if its exploitation is possible to extract useful chemical elements. Specific chemical elements have to be found on the planet for survival (water, oxygen, etc.). They must exist as available local resources or they have to be produced from the exploitation of other local resources.
- Production capacity. The production capacity is defined by the number of items (consumables, tools, …) that can be produced among the list of all items needed for survival for a given period of time. For a given number of settlers, the capacity has to reach an acceptable threshold allowing survival and development of the settlement.

Logically speaking, from an engineering standpoint, survival can be simply expressed as follows (1):

1 $$working\,time\,requirements < working\,time\,capacity$$

requirements and capacities can only be compared for a given period of time. It is proposed here to consider the orbital period of the planet (OP) as the reference time for comparisons. As many objects have a longer lifetime (life support systems, habitation, etc.) the time for their maintenance, repair or reconstruction has to be taken into account using a proportion of the OP. If it is possible to find appropriate resources on the planet, the working time requirements and the working time capacity depend on:

- The number of settlers.
- What living conditions are acceptable.
- The main engineering choices for agriculture, industry and life support.
- The sharing and organization of the settlement.

As the number of people grows, the needs for survival also grow. Nevertheless, as some objects can be shared among several individuals (e.g., a habitat or a vehicle), the working time requirements grow slower than the working time capacity. It is therefore expected that, above a minimum number of individuals, the constraint is satisfied and survival becomes possible.

### Working time capacity

The right hand part of the expression is the working time capacity. “working” is defined here in the broad sense, excluding sleeping, eating, hygiene and resting, but including activities such as raising babies, preparing meals and social activities, which are required for mental health and cohesion of the group. An estimation can easily be provided. The cumulated annual living time is given by the orbital period times the number of individuals. Part of this time is used for sleeping (one third), or for eating, hygiene, and resting (one twelfth). In addition, children and elderly persons are less productive. About one fourth of the population is assumed unproductive. All in all, the annual working time capacity is estimated at 31.25% of the annual living time. See methods for the details.

### Working time requirements

**Figure 1:**
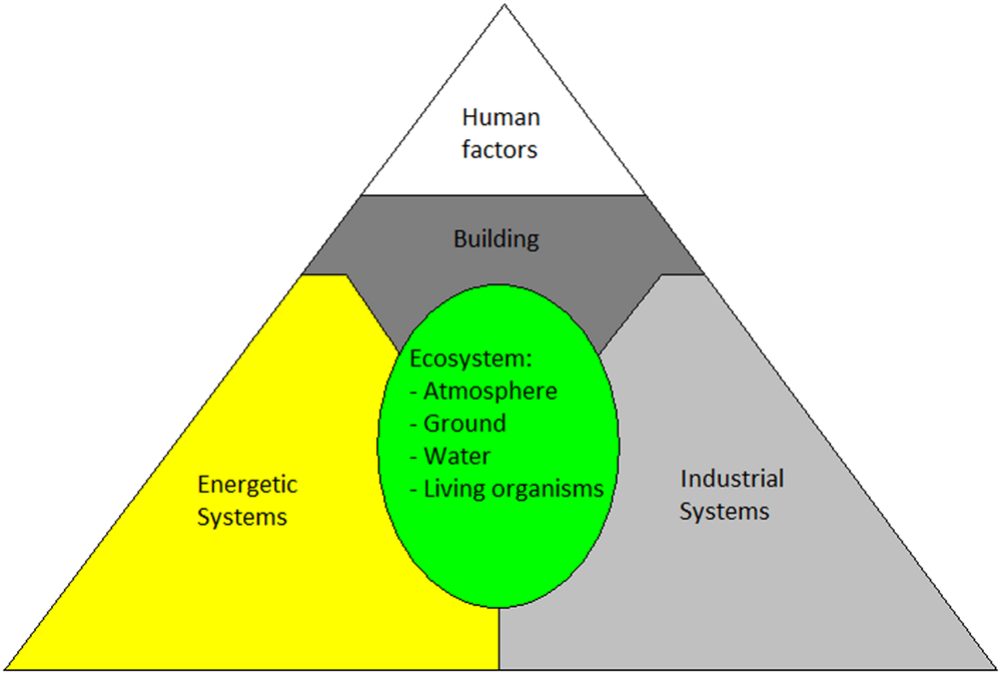
For survival, 5 important domains are highlighted.

The annual working time requirements can be estimated by determining the set of all essential human activities required for survival. Obviously, in order to fulfil fundamental needs such as breathing, eating, or raising children, various industrial processes have to be implemented. For instance, the construction of a new building might require extracting specific ores and the use of tools made of iron, which implies an iron industry. For survival with a short number of individuals, the working time requirements have to be minimized. As a consequence, it is necessary to minimize the number of industries and at the same time to maximize productivity using modern tools. A trade-off must therefore be found between the productivity rate and the small size of the industrial development. As a first approach to the problem, a possible approach is to split human activities into 5 domains, as illustrated Fig. 1:

- d_1_: In the domain of ecosystem management, the main activities are designing and maintaining systems for the production of appropriate gases, controlling air composition, pressure and temperature in habitable modules, collecting, producing and recycling water, controlling life cycles of all living organisms, processing organic wastes, growing plants for agriculture and finally producing and storing food.
- d_2_: In the domain of energy production, depending on the strategy, for instance if it is decided to use photovoltaic cells to produce electricity and to produce methane for vehicles, the main activities are linked to the extraction and processing of silicates, producing photovoltaic cells, wires, and extracting methane thanks to a Sabatier reactor. Heating also belongs in this category.
- d_3_: In the domain of industry, especially metallurgy and chemistry, the main activities are extracting, collecting and processing appropriate ores, making construction materials, manufacturing objects, and producing tools for other activities (e.g., agriculture). Industry may also be concerned in the production of glass, ceramics and plastics, as well as clothes and medicine depending on the strategic choices for survival.
- d_4_: Building domain. Even if a base is built before the arrival of humans, it will have to be frequently reorganized according to the evolution of the settlement (number of settlers, new industries, new organization of the work, new way of life, etc.). In addition, the lifetime of any construction is limited. New habitable zones with new ecosystems and new factories will have to be built close to interesting local resources. Everything will have to be designed and organized for good living conditions and optimization of the work. All activities linked to the architecture, organization (or reorganization), maintenance and construction of buildings are included here.
- d_5_: The last domain concerns social activities. For survival and for the development of the settlement (without development, a small settlement could easily collapse), it is important to raise children and to educate them. It is therefore assumed that the growing rate of the population is positive. Other fundamental human activities concern health care, preparing meals, cleaning, washing, organizing the work and making decisions. For survival, time for sport, culture and entertainment can be minimized, but probably not totally eliminated.

Importantly, for each activity, some margins must be taken into account to cope with unforeseen events. Other activities must concern adaptations of processes to the size of the population and time for reorganization and industrial mutations.

### Sharing factor

As the number of individuals grows in the settlement, more and more objects are shared among them. For instance, people can live in the same habitat, sharing the same air revitalization system, the same water processing system, the same energy production system, etc. If each settler was completely isolated and no sharing was possible, each individual would have to perform all activities and the total time requirement would be obtained by a multiplication by the number of individuals. In addition, a greater number of individuals makes it possible to be more efficient through specialization and to implement other industries allowing the use of more efficient tools. In order to take the possibility of sharing and productivity into account, a sharing factor can be introduced. The time requirement for a given single activity can be simply divided by this factor, which would be different for each activity and for each number of individuals. The sharing factor can be estimated by different functions (polynomial, trigonometric, step functions, etc.). In a restricted range, it can be conveniently estimated by Eq. (2). See Methods for a detailed analysis of the sharing factor.

2 $$s({a}_{i},n)={n}^{{\propto }_{i}}$$

Where:

$${a}_{i}$$: activity i

n: number of settlers

$s({a}_{i},n)$: sharing factor for activity ***i*** with ***n*** the number of settlers

${\propto }_{i}$: constant associated to activity ***i***

Remark: The sharing factor depends on the needs, the processes, the resources and environmental conditions, which may be different depending on the planet.

### Mathematical expression

A mathematical expression of constraint (1) can now be obtained. As the total working time capacity is linearly correlated with the number of individuals and the way of calculating the total time requirement for all activities is also a factor of n, n can be eliminated in both sides of the equation.

The survival of the settlers can finally be determined by Eq. (3).

3 $$\mathop{\sum }\limits_{i=1}^{i={k}_{1}}\frac{r({a}_{1,i})}{s({a}_{1,i},n)}+\mathop{\sum }\limits_{i=1}^{i={k}_{2}}\frac{r({a}_{2,i})}{s({a}_{2,i},n)}+\mathop{\sum }\limits_{i=1}^{i={k}_{3}}\frac{r({a}_{3,i})}{s({a}_{3,i},n)}+\mathop{\sum }\limits_{i=1}^{i={k}_{4}}\frac{r({a}_{4,i})}{s({a}_{4,i},n)}+\mathop{\sum }\limits_{i=1}^{i={k}_{5}}\frac{r({a}_{5,i})}{s({a}_{5,i},n)} < 0.3125\,{\rm{OP}}$$

Where:

$r({a}_{j,i})$ is the individual annual working time requirement to run activity ***i*** in domain ${{\boldsymbol{d}}}_{{\boldsymbol{j}}}$.

$s({a}_{j,i},n)$ is the sharing factor for activity ${{\boldsymbol{a}}}_{{\boldsymbol{j}},{\boldsymbol{i}}}$ with **n** the number of individuals.

*k*_1_ to *k*_5_ are the number of activities for domains ${{\boldsymbol{d}}}_{1}\,$to ${{\boldsymbol{d}}}_{5}$.

The individual annual working time requirement can be estimated for each activity. Sharing factors are thus the only parameters defined as functions of ***n*** (Eq. (2)). The minimum number of settlers for survival is therefore the minimum value of ***n*** for which constraint (3) is respected.

### Risks issues

Even if there are enough individuals for the production of goods, a small society can collapse for many different reasons. According to Marin and Beluffi, these reasons can be infertility, inbreeding, sudden deaths (genetic issues included^7^), accidents or random events^6^. Other important risks with severe impacts concern the loss of important assets, the loss of large amounts of resources, mortal fights among different groups of individuals, or a significant loss of efficiency at work due to inappropriate social organization (e.g., loss of know-how, loss of motivation, bureaucracy, etc.). In order to mitigate the risks, a possible option is to produce more than the minimum. This is especially true at the beginning of the settlement, as any accident could dramatically reduce the production capacity. In the early stages, the severity of the risks also depends on the initial state. In order to mitigate the risks, it will therefore be important to start with large amounts of resources and spare parts. Complementary studies are needed using simulations with different parameters to assess the probability of success for the proposed minimum number of settlers.

## Case Study: Survival on Mars

### Hypotheses

The method is applied to the survival on Mars. The objective is to determine the minimum number of individuals that is compatible with constraint (3). The difficulty is to determine the best trade-off among the different parameters, especially the types of resources that will be exploited, the industries that will be implemented, the organization of the work, the way of life, etc. The specific utilization of Martian resources for life support, agriculture and industrial production has been studied in different workshops and published in reports and books^12–15^. As the complexity of the problem is high, the viability and optimality of any set of choices will remain uncertain. In a recent contest organized by the Mars Society, people were asked to define a realistic scenario to settle the red planet. As our proposal was based on conservative assumptions and the ability to survive if the help from Earthlings were stopped, it is proposed here to use it as a guideline^15^. See Methods for more details about our hypotheses.

### Results

**Figure 2:**
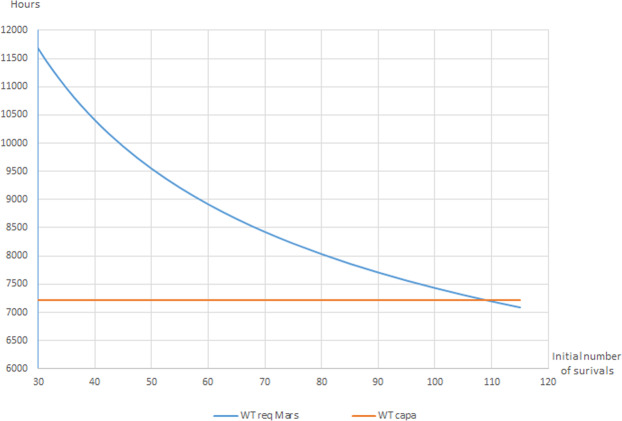
Determination of the minimum number of individuals for survival on Mars. The annual working time capacity is greater than the annual working time requirement if the initial number of individuals is greater than 110.

The total working time requirement per individual to implement each activity has been calculated as a function of the number of individuals. According to Eq. (3), survival is possible only if the working time requirement is less than the working time capacity. As the working time requirement per individual is decreasing with the number of individuals (thanks to sharing factors), the determination of the minimum number of individuals for survival is straightforward. The result is presented Fig. 2. The minimum number of individuals for survival on Mars is 110.

**Figure 3:**
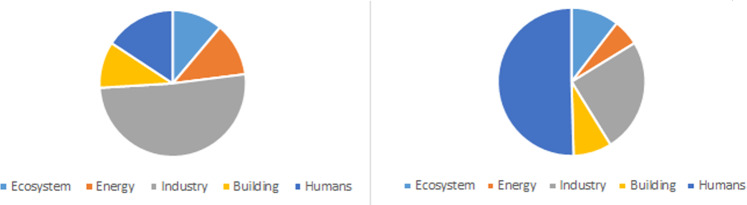
Working time requirement distribution for 1 (left) and 110 individuals (right).

Another interesting result is the distribution of the working time requirement for one individual in comparison with the individual time requirement for 110 individuals (see Fig. 3). For a single person, the problem is to implement all industrial activities, while for 110 persons, half of the working time requirement is in the domain of social activities, including raising babies, health care and cultural events.

## Conclusion

An original method has been proposed to determine the minimum number of individuals for survival on another planet or in space, using Mars as the example. It is based on the comparison between the required working time to fulfil all the needs for survival and the working time capacity of the individuals. An important parameter of the model is the sharing factor, which is used to take sharing and productivity into account. In the example provided to illustrate the method, the estimated minimum number of individuals for survival on Mars is 110. This is obviously a rough estimate with numerous assumptions and uncertainties. To our knowledge, it is nevertheless the first quantitative assessment of the minimum number of individuals for survival based on engineering constraints. Other assumptions can be made. It is also possible to consider the case of survival on the Moon, with different industrial processes, different energy systems, difference life support systems etc. and to compare the results with survival on Mars. Noticeably, as Moon nights last about 14 days, growing plants might require full artificial lighting, which in turn would have a tremendous impact on energy requirements, suggesting that more human resources would be needed for that domain. 110 individuals could thus be insufficient for survival on the Moon but a more detailed study is needed before drawing any conclusion. Our method allows simple comparisons, opening the debate for the best strategy for survival and the best place to succeed.

## Methods

### Annual working time capacity

The annual living time depends on the orbital period of the planet. It is 8,766 hours on Earth and 16, 487 hours on Mars (686.97 × 24). According to the National Sleep Foundation, adults are sleeping 7 to 9 hours on average, which represent about 33% of the living time. On another planet, with a different rotation period, the sleeping time could be different. As no data is available for that, it is assumed here that the sleeping time remains 33% of the living time. Part of the living time also has to be spent for eating and another part for hygiene. In total, on Earth, it can be assumed that approximately 10 hours out of 24 are dedicated to these activities, sleeping included, which represent 41.7% of the living time. Even if the duration of the day is longer or shorter, the same ratio is assumed in first approximation. The presence of children, elderly persons and eventually handicapped persons will reduce the working time capacity. The impact highly depends on the age pyramid. The best strategy for the proportion of children and the shape of the age pyramid at the beginning of the survival time is hard to define. In agreement with the study from Marin and Belufi, it is assumed that the age pyramid must be rather homogeneous to avoid future gaps in productive age classes and too many individuals in the young or elderly age^6^. As a consequence, the number of children in the age range 0 to 10 should be stabilized around 20% of the total. At the age of approximately 10, even if they still need education, children can help adults in many ways and become productive. As a first approximation, the unproductive 20% and the reduction of the productivity for young adults and elderly persons can be taken into account in the working time capacity by removing 25% of the individuals in the total working time capacity. The annual working time capacity is thus estimated at 31.25% of the annual living time (41.7 × 0.75) times the number of individuals. It is important to notice that the specific time required for raising and educating children also has to be estimated but this is accounted for in the working time requirements.

### Sharing factor estimation

The settlers can share the same air revitalization system, the same water processing system, the same habitat, etc. Obviously, the scale of the systems has to be adapted to the number of individuals, but scaling up has often a small impact on the required time to develop and operate the systems. In addition, time savings are expected for a greater number of individuals thanks to specialization and productivity gains. For some activities, however, scaling up does not provide substantial time savings. For instance, the required time for picking fruits on plants is closely correlated with the amount of fruits to be collected, which is linearly correlated to the number of individuals. An important issue is to be able to calculate the sharing factor as a function of the number of individuals. If such a function were available, the minimum number of settlers for survival could be deducted from Eq. (3). Importantly, whatever the activity, the sharing factor is equal to 1 for 1 individual and then increases with the number of individuals according to a curve that follows, as a first approximation and in most cases, a logarithmic law (in a way similar to productivity gains). Such curves can be conveniently approximated by Eq. (2) with $\propto$ in the range [0;1].

Sometimes, this depends on the organization of the society. Imagine for instance that the settlement is split into groups of a hundred individuals with autonomous life support and energy systems. In this case, the sharing factor of activities linked to life support or energy would resemble a step function, with a monotonic growing rate below 100 and a division by a factor of 2 when 101 is reached, then another monotonic rate, etc. The most appropriate function must be used if it is known. For the sake of simplicity, and in the context of this preliminary study, it is suggested here to use Eq. (2) to estimate the sharing factor of most activities. In order to determine ${\propto }_{i}$ the proposed method is to interpolate the function with two points. The first point is given with n=1 by ${1}^{{\propto }_{i}}=1$ and the second point is given by an estimation of the sharing factor for 100 individuals. Then ${\propto }_{i}$ is chosen such that ${100}^{{\propto }_{i}}$ is a good approximation of that value.

Noticeably, if the activity is completely modified for a greater number of individuals due to adaptations of technologies and procedures, it is possible to take changes into account by an appropriate modification of the sharing factor.

Let us illustrate this method with 2 examples:

- The same oxygen production unit (based on water electrolysis) can be shared by all individuals of the same habitable area. The scale of that unit must be adapted to the production needs, but the sharing factor quickly increases with the number of individuals. At some point, however, other oxygen production units must be built for other habitable areas. The sharing is not increasing any more, but there is an industrial development allowing the use of better materials and more efficient processes. For large values of n, productivity gains are therefore expected and the sharing factor can still be used to take these gains into account. For 100 individuals, the sharing factor is estimated at 25. For oxygen production and n individuals, a good estimate is thus provided by ${n}^{0.7}$.
- For the construction of a new habitat, 10 individuals specialized in an activity may work 20 times faster than a single person doing everything. For 10 individuals, the total working time would therefore be divided by a sharing factor of 2. Now, in order to divide the working time by another factor of 2, the number of individuals might have to exceed 100. In this case, ${n}^{0.3}\,$would be a good approximation of the sharing factor.

### Main hypotheses for survival on Mars

Our proposal is based on a previous study, which aimed at defining the best way to implement a Martian settlement^15^. The survival capability, which was considered essential, was based on three important principles:

- Make it simple: In the initial state, all individuals will live under a dome and will share the same life support system pressurized at 350 mb with 80% oxygen. Plants will be grown in numerous greenhouses made of glass maintained in an iron based structure with reflectors behind to provide sufficient light. In order to create an appropriate soil for plants, it will be necessary to make a mixed of rocks, salts, water and to add also organic wastes and decomposers (insects and microorganisms). Water will be extracted from icy terrain and recycled using natural filters. The idea is to minimize the need for complex objects. There will be no LED for artificial lighting, there will be no complex chemistry for hydroponic cultures and water will be filtered without artificial systems. For energy, however, it is not possible to avoid artificial systems. Photovoltaic cells will provide electricity, biomass (methane) will be used to power engines and solar furnaces will be used to heat up materials. Though rather limited, several important industries will have to be implemented to produce iron based objects (including simple vehicles), clothes (there must exist a way to make a space suit from available local resources), glass, ceramics, duricrete (concrete like material) and photovoltaic cells. An important issue is to determine if modern tools are necessary for survival on Mars and if global productivity gains enabled by computers, robots and other modern tools, are greater than the additional time requirements for their production and maintenance. The production of photovoltaic cells and spacesuits, for examples, will certainly be difficult technical challenges if no modern tool is used. It is nevertheless assumed here that such challenges can be faced and that all activities for survival are possible without computers and robots. For radiation shielding, it is generally recommended to add a few meters of regolith above the habitat to reduce radiation levels below acceptable limits (on Earth, it is 100 REM in 5 years)^13,14^. However, above official limits, the probability of dying from cancer due to radiations is slowly increasing. Full shielding is a possible option, but survivors might choose a tradeoff between no shielding and full shielding in order to get good living conditions (sunny lighting with transparent windows) perhaps at the expense of 3 to 5 years of average life expectancy.
- Maximize sharing: The group of survivors can share the dome’s ecosystem - air, water, food, energy - and also recycling systems - tools, spacesuits, vehicles, industries, etc.
- Develop step by step: For short term survival, it is possible to accept harsh living conditions, but it is not acceptable in the long term. The evolution of the settlement can be made possible by first accumulating resources (human resources, ores, wood, iron, backup food, backup tools, etc.) and then creating a new base elsewhere with volunteers. The accumulation of resources will compensate for the temporary lack of human resources. Then for each new base, efforts can be concentrated on the development of new industries in order to achieve a modern society step by step, after centuries of development.

See^15^ for more details about the settlement strategy. The list of human activities required for survival can be deduced from the proposed strategy in each of the five domains that have been identified and are illustrated Fig. 1. The time requirement and sharing factor for each activity can then be estimated as a function of n, defined as the number of individuals.

### Estimation of time requirements for survival on Mars

The main activities and their time requirements are listed Tables 1, 2, 3, 4 and 5 for ecosystem management, energy production, industry production, building construction, and social activities, respectively. For each activity, the annual working time requirement per individual has been estimated with associated range for 95% confidence interval (heuristically determined), as well as the sharing factor according to the number of individuals, taking sharing and productivity gains into account.

- Ecosystem management: Agriculture is probably the activity that requires more time than the others when the size of the group is high. The reason is because the needs (kilograms of vegetables, fruit, etc.) will increase linearly with the number of individuals and the sharing factor is therefore relatively weak and linked only to productivity gains. Water management takes a lot of time, but the same system can be shared by many people, which means that the sharing factor is very high. See Table 1.

- Energy domain: Photovoltaic cells will not have to be produced all the time, but as they will have to be changed after about fifteen years, the average annual production rate will not be negligible. For electricity, some electronic devices and wires will have to be produced. For such activities, it is assumed that industrial assets will provide basic elements such as iron, silicon, copper, plastics, etc., or appropriate substitutes. Even if energy consumption is highly correlated with the number of individuals, a large part of that energy will be used to produce objects that will be shared (for instance vehicles, tools, industrial ovens, etc.). It is therefore assumed that the sharing factor is quickly increasing with n. See Table 2.

- Industrial domain: Rich metallic ores may be hard to find on Mars. However, even if extraction is not as efficient as it is on Earth, some resources can easily be exploited. Hematite is for example present and can be processed to obtain ferrous elements. Silicates can also be found in the sand of dunes. Producing glass and photovoltaic cells from it will not be easy, but it is feasible. Eventually, a trade-off can be found between purity and complexity of chemical processes. A lot of time is allocated to industrial activities, from mining to metal production and object manufacturing. Numerous objects will indeed be used in other domains, for instance pumps for agriculture, wires for electricity, tools for mining, metallic structures to support the walls of buildings, textiles for space suits, etc. The positive point is that a lot of time can be saved when n increases by sharing and also by means of productivity gains.

- Building domain: Even if it is assumed that a base has been built prior to the arrival of the survivals group, many systems will require regular maintenance and, after two or three decades, the walls, the roof and the structure of the base may suffer from cracks and degradation. For long term survival, it is imperative for the group to be able to build new bases, including greenhouses, habitats, small factories, etc. The average annual working time may not be high, and many buildings can be shared, but it is not negligible.

- Social activities: Let us consider first raising babies and young children. This activity involves many different elementary activities such as feeding, washing, dressing, watching, etc. As the babies’ ratio must be constant, as a first approximation, feeding them requires a time that is linearly correlated with the number of settlers. For this activity, the sharing factor would therefore be close to 1 (no sharing). However, watching babies and young children while they are playing or sleeping could be carried out in parallel with a constant ratio of 1 person for 20 of them (certainly less when they are very young and more when they are getting older). The sharing factor would thus be equal to 20 at the maximum, for large values of n. On average, taking all elementary activities for raising babies into account, it is assumed that the sharing factor slowly increases towards 8, which would be an asymptotic maximum. If we set SF(1) = 1 and SF(100) = 4, n^0.3^ would be a good approximation of the sharing factor for small values of n but not for large values. A trade-off is proposed: if n < 1024 SF(n) = n^0.3^ otherwise SF(n) = 8. Apart from raising children and education, many other activities fall into this domain. First, health care, including teeth washing, hand washing, haircuts, etc. has to be taken into account. The preparation of meals also takes some time. It is assumed here that the group will share such preparation to save time. Another important issue is to take cultural events, sports and entertainment into account. For short term survival, it is possible to work every hour of the day during dozens of days, but for the long term, it does not sound a reasonable assumption. It is assumed here that one twelfth of the living time will be dedicated to these unproductive but necessary activities to strengthen the mental health of each individual and social cohesion. Finally, it is also important to consider some time for the organization of the group, especially talking about problems, decision making, reorganization of the work, and also for possible innovations, which will probably be necessary and fairly frequent.

**Table 1:** Time requirements in the domain of ecosystem management.

Activity for ecosystem management	Annual time requirement per individual with range for 95% CI	Sharing factor as a function of n with range for 95% CI
Agriculture	940 ± 280	n^0.3 ±0.05^
Air management	380 ± 110	n^0.7 ±0.1^
Water management	750 ± 220	n^0.7 ±0.1^
Living organisms management	940 ± 280	n^0.5 ±0.08^
Organic wastes management	940 ± 280	n^0.5 ±0.08^
Agronomy	940 ± 280	n^0.4 ±0.1^
Other activities, including innovation	1880 ± 1000	n^0.5 ±0.2^

**Table 2:** Time requirements in the domain of energy.

Activity for energy production and management	Annual time requirement per individual with range for 95% CI	Sharing factor as a function of n with range for 95% CI
Photovoltaic cells production	1880 ± 750	n^0.6 ±0.15^
Electricity management	1880 ± 940	n^0.6 ±0.15^
Thermal management	380 ± 110	n^0.6 ±0.15^
Methane production and storage	940 ± 280	n^0.6 ±0.15^
Other activities, including innovation	1880 ± 1000	n^0.6 ±0.25^

**Table 3:** Time requirements in the domain of industry.

Industrial activity	Annual time requirement per individual with range for 95% CI	Sharing factor as a function of n with range for 95% CI
Mining (silicates, metallic ores, …)	3802 ± 1900	n^0.6 ±0.15^
Metal production	5640 ± 3800	n^0.6 ±0.15^
Production of metallic objects	5640 ± 3800	n^0.6 ±0.15^
Ceramics and glass production	1880 ± 940	n^0.6 ±0.15^
Chemical industry	5640 ± 3800	n^0.6 ±0.2^
Production of clothes	1880 ± 940	n^0.6 ±0.2^
Other activities, including innovation	5640 ± 3800	n^0.6 ±0.2^

**Table 4:** Time requirements in the domain of building.

Activity in the building industry	Annual time requirement per individual with range for 95% CI	Sharing factor as a function of n with range for 95% CI
Production of concrete or equivalent	380 ± 110	n^0.7 ±0.2^
Construction of new buildings	940 ± 280	n^0.7 ±0.2^
Equipping new buildings	940 ± 280	n^0.5 ±0.15^
Maintenance	1880 ± 470	n^0.4 ±0.1^
Other activities, including innovation	1880 ± 940	n^0.5 ±0.2^

**Table 5:** Time requirements in the domain of social activities.

Social activities	Annual time requirement per individual	Sharing factor as a function of n
Raising babies	1500	n < 1024, n^0.3^ n ≥ 1024, n = 8
Education and training	1500	n^0.6^
Health care and medical acts	1500	n^0.1^
Meal preparation	560	n^0.6^
Social organization and innovation	940	n^0.2^
, sports, entertainment	940	1
Other activities, including innovation	1880	n^0.3^

All values are integrated in Eq. (3). Then, the total annual time requirement is calculated as a function of n. The minimum value of n for which the total time requirement is below the threshold (working time capacity) is the minimum number of individuals for survival.

## References

[1] Bostrom, N., Existential Risks Analyzing Human Extinction Scenarios and Related Hazards, *Journal of Evolution and Technology* **9** (1), (2002).

[2] Sagan, C. Pale Blue Dot: A Vision of the Human Future in Space (1994).

[3] Moore, J. H. Kin-based crews for interstellar multi-generational space travel, in: K. Yoji, F. Bruhweiler, J. Moore, C. Sheffield (Eds.), Interstellar Travel and Multi-Generation Space Ships, Collectors Guide Publishing, Burlington, Ontario, Canada, 80–88 (2003).

[4] Smith CM (2014). Estimation of a genetically viable population for multigenerational interstellar voyaging: Review and data for project Hyperion. Acta Astronautica.

[5] Smith, C. M., An Adaptive Paradigm for Human Space Settlement, *Acta Astronautica* **119**, (November 2015).

[6] Marin F, Beluffi C (2018). Computing the minimal crew for a multi-generational space journey towards Proxima Centauri b. Journal of the British Interplanetary Society (JBIS).

[7] Lynch, M., Conery, J., Reinhard0, B., Mutational Meltdowns in Sexual Populations, Evolution **49**(6), 1067–1080 (1995). 10.1111/j.1558-5646.1995.tb04434.x 28568521

[8] Drake, G., ed., Mars Architecture Steering Group, 1^st^ Addendum of the Human Exploration of Mars, Design Reference Architecture 5.0, NASA Johnson Space Center (2009).

[9] Drake, G. ed., Mars Architecture Steering Group, 2^nd^ Addendum of the Human Exploration of Mars, Design Reference Architecture 5.0, NASA Johnson Space Center (2014).

[10] Genta, G. & Salotti, J.-M.(ed.), Global Human Mars System Missions Exploration, Goals, Requirements and Technologies, *Cosmic Study of the International Academy of Astronautics*, (January 2016).

[11] Salotti, J.-M. Robust, affordable, semi-direct Mars mission, *Acta Astronautica* **127**, October–November, 235–248, (2016).

[12] Johnson, R. D. & Holbrow, C., Space Settlements – A Design Study, NASA Scientific and Technical Information Office. SP-413 (1977).

[13] Zubrin, R. & Wagner, R. The Case for Mars: The Plan to Settle the Red Planet and Why We Must, ISBN 978-0684835501 (2011).

[14] Petrov, G. I., Mackenzie, B., Homnick, M. & Palai, J., A Permanent Settlement on Mars: The Architecture of the Mars Homestead Project”, proc. of the International Conference On Environmental Systems, Roma, Italy (July 2005).

[15] Salotti, J.-M. Chapter 5: The Team ENSC Vizzavona Design, in Mars Colonies: Plans for Settling the Red Planet, Frank Crossman ed., Polaris Books, Lakewood, Colorado (2019).

[16] O’Neill, G. K., The Colonization of Space, *Physics Today* **27**(9), 32–40, (September 1974).

[17] Matloff GL (1976). Utilization of O’Neill Model I Lagrange Point Colony as an Interstellar Ark. Journal of the British Interplanetary Society (JBIS).

[18] Bond A, Martin AR (1984). World Ships – An Assessment of the Engineering Feasibility”. Journal of the British Interplanetary Society (JBIS).

[19] Hein AM, Pak M, Pütz D, Bühler C, Reiss P (2012). World Ships, Architectures & Feasibility Revisited”. Journal of the British Interplanetary Society (JBIS).

